# Supplementary material for: Metabolic Dynamics of In Vitro CD8+ T Cell Activation
Source: Metabolites. 2020 Dec 28;11(1):12. doi: 10.3390/metabo11010012 (PMC7823996; doi:10.3390/metabo11010012)
Supplement: Supplementary file 1 [file metabolites-11-00012-s001.zip › metabolites-991822-supplement/Supplementary material.pdf]

Supplementary Material
to

Metabolic dynamics of in vitro CD8+ T cell activation

by

Joy Edwards-Hicks, Michael Mitterer, Erika L Pearce, Joerg M Buescher

List of tables in Excel format

Supp_TableS1_FIA_neg.xlsx	Feature table of negative mode FIA analysis
Supp_TableS2_FIA_pos.xlsx	Feature table of positive mode FIA analysis
Supp_TableS3_ HILIC_neg.xlsx	Feature table of LC-MS analysis using HILIC chromatography and negative ionization mode
Supp_TableS4_polarRP_pos.xlsx	Feature table of LC-MS analysis using reverse phase chromatography and positive ionization mode
Supp_TableS5_lipid_neg.xlsx	Feature table of LC-MS analysis using lipid chromatography and negative ionization mode
Supp_TableS6_lipid_pos.xlsx	Feature table of LC-MS analysis using lipid chromatography and positive ionization mode
Supp_TableS7_PCA.xlsx	Scores and loadings of principle component analysis of combined polar data set

Supplementary Figure S1

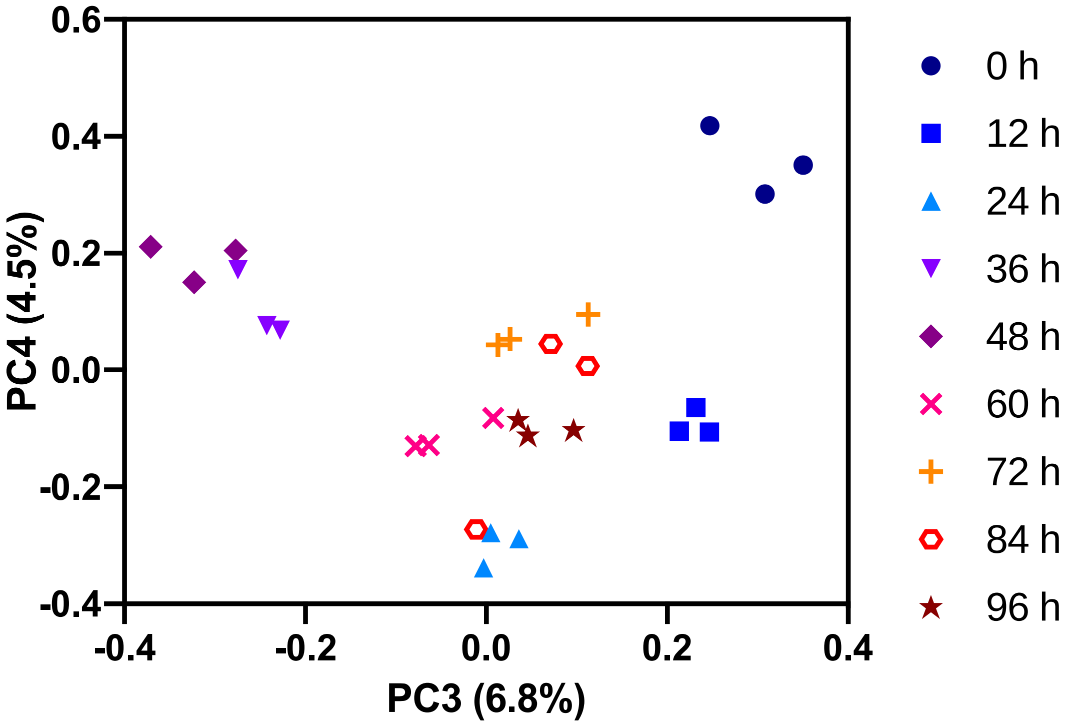

Principal component (PC) 3 and 4 of principle component analysis of combined polar data sets show separation of replicates. These PCs probably reflect more subtle metabolic changes occurring during the activation of TN to TE.

Supplementary Figure S2

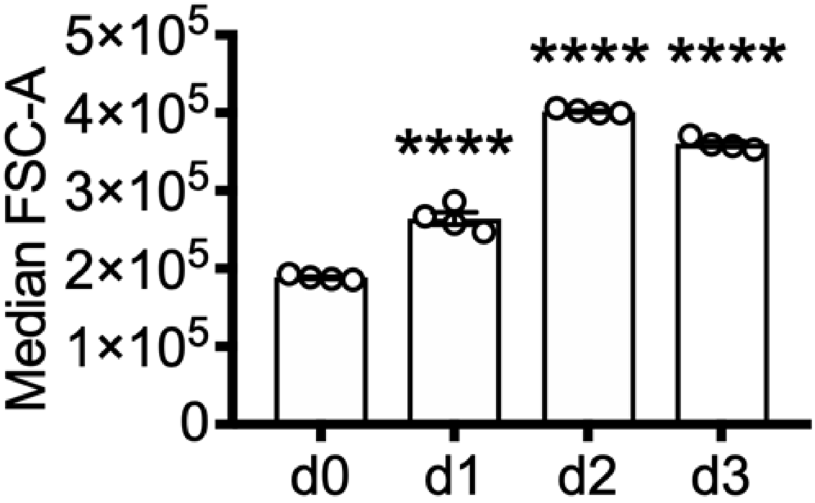

Size of CD8+ T-cells measured as forward scatter by FACS show an increase in cell size following in vitro activation from TN to TE. X-axis is time in days.

Supplementary Figure S3

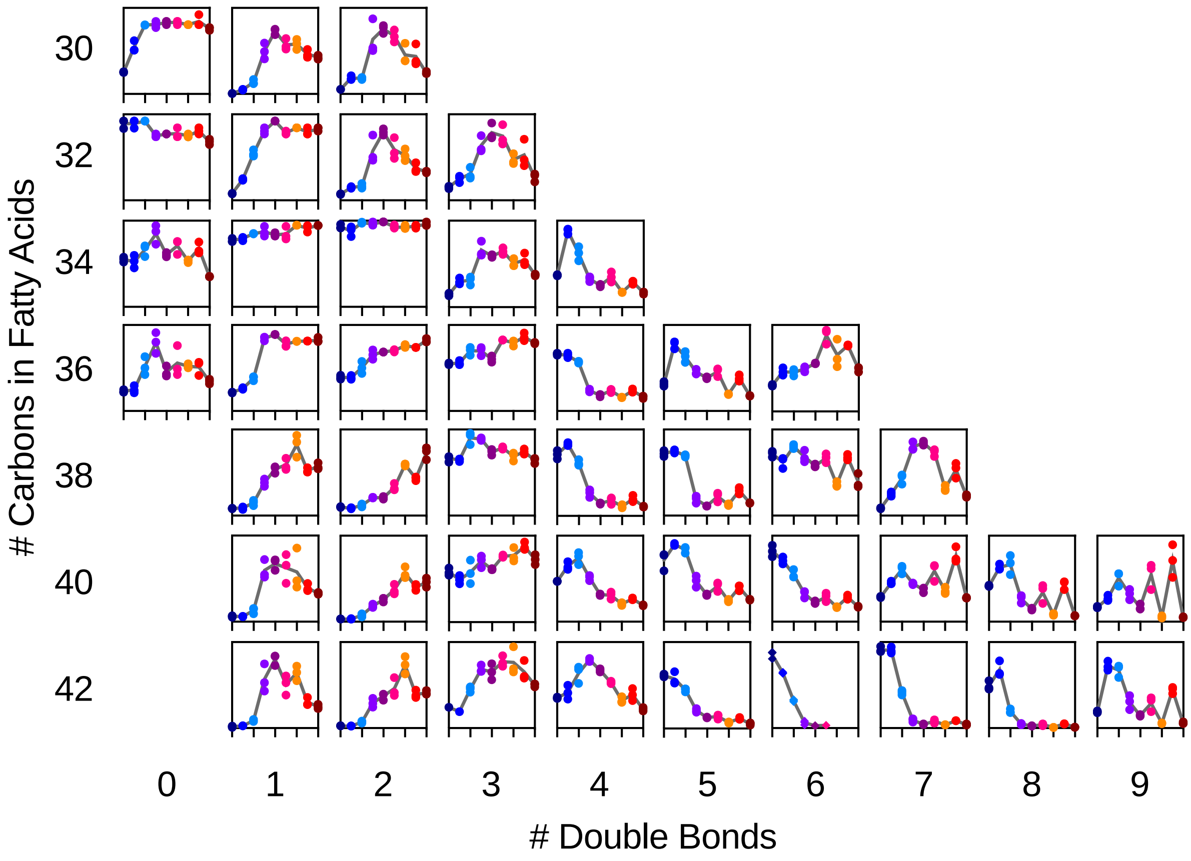

Time courses of phosphatidyl choline species plotted on a grid of number of carbons in acyl chains (vertically) and number of double bonds in acyl chains (horizontally). Each sub-plot shows time on the x-axis and signal intensity on the y-axis.
